# The Road Not Taken: Misclassifying an Anti‐Seizure Medication as a Failure

**DOI:** 10.1002/acn3.70139

**Published:** 2025-07-21

**Authors:** Christopher N. Henry, Daniel M. Goldenholz

**Affiliations:** ^1^ Department of Neurology Children's Hospital of Richmond at VCU Health Virginia United States; ^2^ Beth Israel Deaconess Medical Center, Department of Neurology United States; ^3^ Harvard Medical School United States

**Keywords:** anti‐seizure medication, bioinformatics, epilepsy, simulation, statistics

## Abstract

**Objective:**

To quantify how often anti‐seizure medications (ASMs) appear ineffective yet provide benefit when considering seizure frequency (SF) variability.

**Methods:**

We used the CHOCOLATES seizure diary simulator to generate 100,000 patient seizure diaries that reflect natural SF variation in a heterogeneous population. Medication effect was modeled as a 20% average SF reduction (standard deviation 10%). We identified how many patients with an observed ≥ 25% SF increase (apparent worsening) actually had a true ≥ 10% SF reduction (vs. no medication), and how many with an observed ≥ 50% SF reduction (apparent responders) would have shown < 0% reduction if not taking the ASM. We also quantified how many individuals who had apparent worsening were actual worsening (> 0% SF increase vs. no medication).

**Results:**

Simulations closely matched real‐world ASM trials, showing a median SF reduction of 36% with ASM versus 17% with placebo; 35% of patients on ASM achieved ≥ 50% SF reduction versus 20% on placebo. Apparent worsening occurred in 12%; among these, 76% were true improvers. Of the apparent responders, 12% were true nonresponders. Only 4% of the individuals with apparent worsening truly worsened compared to no medication.

**Interpretation:**

SF variability can lead to significant misclassification of ASM benefit. Many patients labeled as having “failed” an ASM trial were likely receiving meaningful benefit and may warrant reconsideration of the medication. Prospective clinical studies are needed to determine how best to account for SF variability and refine the interpretation of treatment response in epilepsy management.

## 1 Introduction

People with epilepsy fail anti‐seizure medication (ASM) due to a lack of effectiveness, side effects, or both. As patients fail successive ASMs, their therapeutic options become increasingly limited, often forcing a challenging trade‐off between the degree of seizure control and the burden of adverse effects. For example, in Lennox–Gastaut Syndrome (LGS), patients will have tried an average of 7–8 ASMs [1]. Therefore, misclassification due to lack of effectiveness can have significant clinical implications. In this study, we aimed to quantify how often such misclassification can occur due to natural variability in seizure frequency (SF).

**FIGURE 1:**
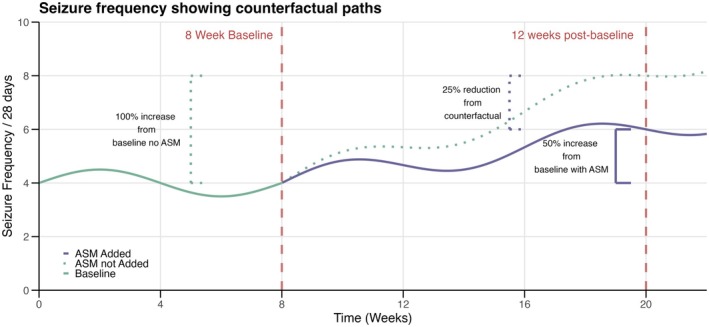
A hypothetical patient is given an adjuvant anti‐seizure medication (ASM) after 8 weeks of baseline measurement. The purple line is the cumulative seizure frequency on medication. The green dotted line is the cumulative seizure frequency without the new ASM. This patient experienced a 50% increase in seizure frequency from baseline, but would have had a 100% increase if they were not taking the ASM (the counterfactual scenario). Therefore, the medication decreased the patient's seizure frequency by 25% from counterfactual. Unfortunately, real patients never know how much reduction they experience compared to the counterfactual. This is one of the advantages of synthetic patients—we can simulate reality and the counterfactual.

SF is known to vary over time, independent of treatment interventions [2]. Recognizing the potential for natural SF fluctuations to impact assessments of ASM efficacy is imperative. (Figure 1) shows how an individual prescribed an adjunctive ASM might experience a 50% increase from a baseline of 4 seizure/month to 6 seizure/month, but would have had a 100% increase to 8 seizure/month if they did not start the new ASM (counterfactual scenario).

There is indirect evidence from regulatory adjunctive ASM trials that SF variability may lead to misclassifying individuals as having failed an ASM due to worsening SF. In a study that looked at all regulatory trials from 2000–2023, between 19% and 50% of the participants randomized to placebo showed an increase in SF (apparent worsening). Conversely, the proportion of patients with apparent worsening was consistently smaller in the ASM group (5%–37%) [3]. These findings suggest that SF increases often reflect natural variability rather than medication failure.

In this study, we generated synthetic data to explore the role of natural fluctuations in creating the misperception of drug failure. Given synthetic data, we were able to generate both a drug situation and the no‐drug situation in the same patient—as if we had access to the hypothetical parallel universe where the same patient was not taking the medicine during that same observation period. We refer to this hypothetical as the “counterfactual”—meaning the alternative reality without the drug. We hypothesized natural fluctuations in SF can lead some patients to experience a worsening of seizures despite receiving a benefit.

## 2 Methods

We used the CHOCOLATES seizure diary simulator to generate 100,000 patient seizure diaries that simulate multiple aspects of natural SF variation [4]. We then included patients based on commonly used SF inclusion criteria in regulatory trials. This allowed us to check that our model was producing SF distributions seen among real patients. Similar to trials, we used an 8‐week baseline measurement and patients had to have no fewer than 8 seizures during that time and no seizure‐free period longer than 21 days [5].

The CHOCOLATES software does not require any data input at all. It was developed based on the statistical features of multiple previously published datasets [4]. By design, CHOCOLATES will generate each patient with a unique seizure frequency such that if a population were generated, then the population would look similar to previously studied populations. Additionally, each synthetic seizure diary has the potential to include clustering, seizure risk cycles, and no more than a maximum number of seizures per day, as specified by the CHOCOLATES model.

We then applied a Gaussian distributed heterogeneous (across patients) medication effect with a mean of 20% reduction and a 10% standard deviation to the SF during the treatment period (week 9–week 20). The standard deviation allowed for the ASM to cause SF increases (“true worsening”) in a small fraction of the patients, while the majority of patients would experience SF reductions (“true improvement”). Other medication effect models were considered (see Appendix Data S1). To ensure our trial eligible synthetic patients behaved similarly to real‐world trial patients, we generated 10,000 randomized trials. From the eligible pool of patients, we randomly selected 400 unique patients without replacement within each trial. This ensured that within any single trial, no patient appeared more than once. However, because patients were sampled independently for each trial, a patient could be included in multiple trials across the simulations. Selected patients were randomly assigned to either the ASM group or the placebo group with equal probability. Randomization was performed independently within each trial. We cross‐referenced the median percent reduction and 50% responder rate to results in regulatory trials.

We then quantified how many of the trial eligible patients with at least a 25% increase in SF from baseline (“apparent worsening”) had a reduction equal to or greater than 10% from the SF they would have had without the ASM (“true improvement”). We chose a 25% increase as a significant worsening based on its use in other trials, but acknowledge that the definition varies from trial to trial [5, 6, 7]. We used 10% as the minimum effective threshold based on the median percent reduction over placebo observed with 1000 mg/day of levetiracetam in regulatory trials for adjunctive therapy approval [8]. To contextualize the misclassification of responder versus failure, we quantified how many individuals with an observed > 50% reduction in SF (“apparent responders”) received less than 10% SF reduction compared to the counterfactual (“true nonresponders”). Lastly, we quantified how many individuals with ≥ 25% SF increase (“apparent worseners”) were actually harmed by the ASM, meaning the ASM caused > 0% increase in SF from the counterfactual (“true worseners”).

Our simulations were conducted using R (version 4.4.0) as well as Python (v 3.12.4). We utilized ChatGPT‐4.o (OpenAI, California, USA) to draft an initial coding framework and to debug portions of the R code. The tool provided assistance intermittently between July and December 2024. C.N.H. verified that the code was accurate and appropriately handled the data to ensure the reliability of the results. Open source code is available at https://github.com/forkcandles/misalignment_project.

## 3 Results

**FIGURE 2:**
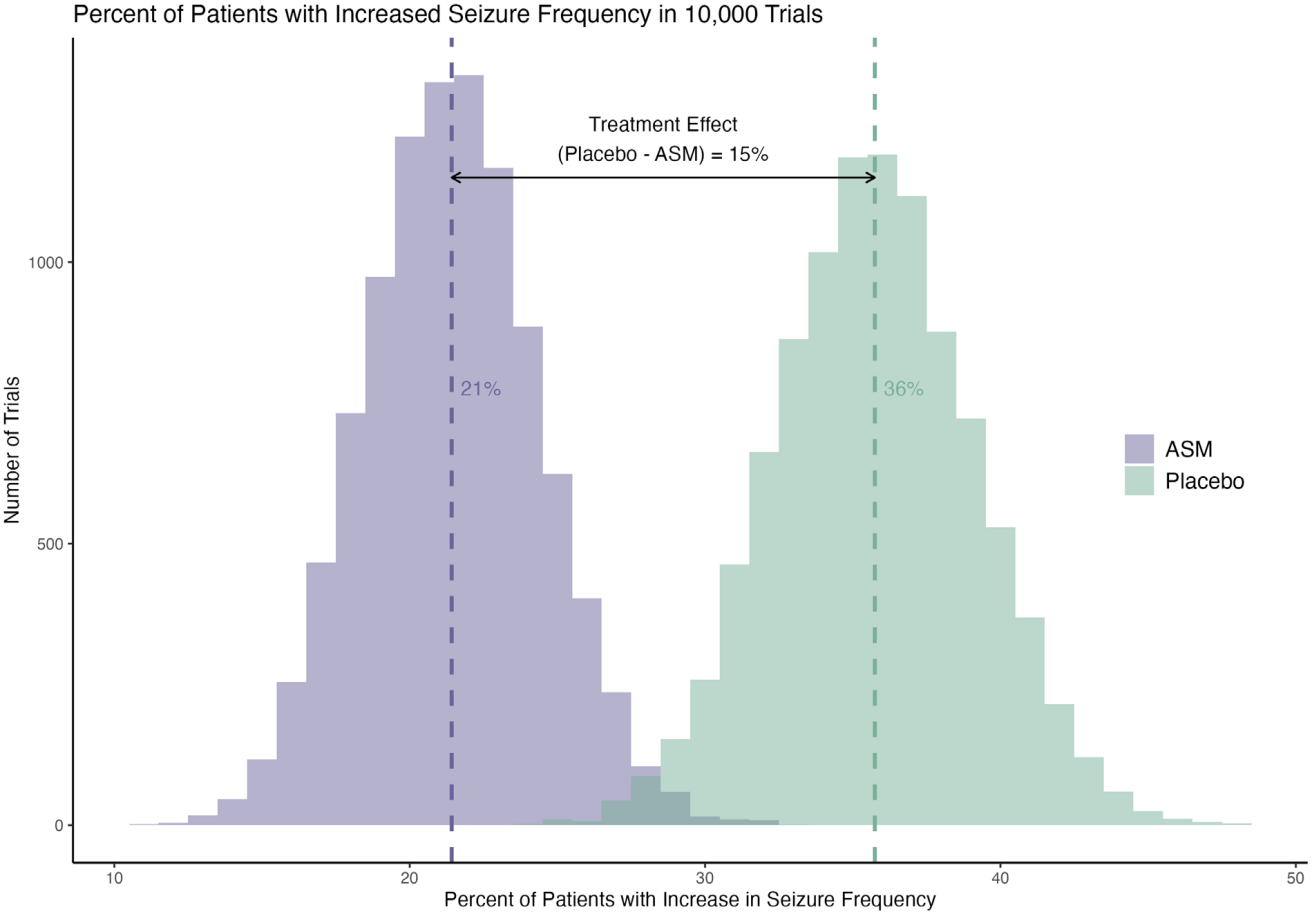
The percentage of patients with an increase of any degree (> 0% increase) in each of 10,000 simulated trials with an N of 200 in the ASM and placebo group. The mean and distribution was consistent with those reported in adjunctive ASM regulatory studies of this size. There was 15% less patients with an increase when taking an ASM compared to not taking the ASM.

**FIGURE 3:**
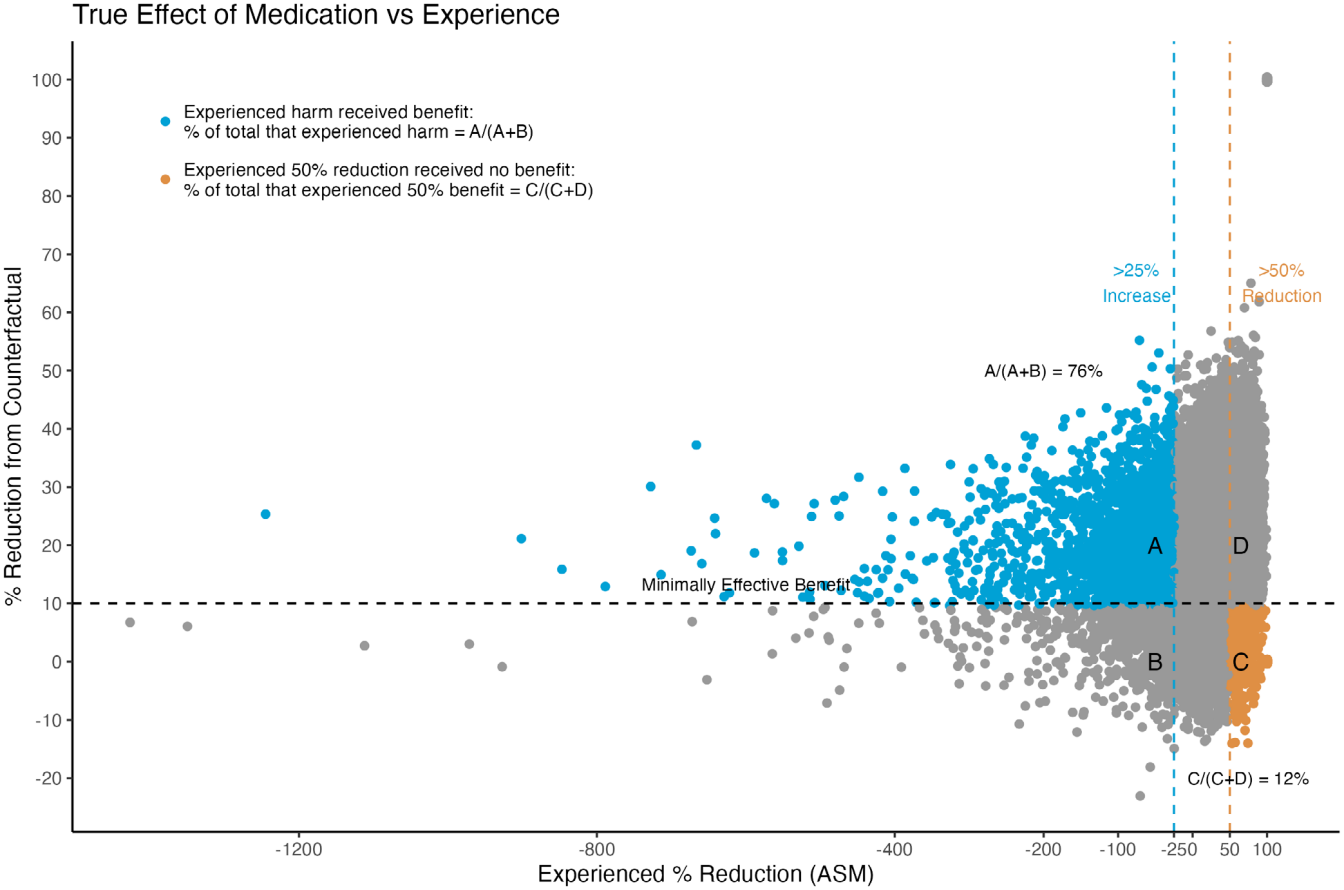
Relationship between true medication effect and experienced benefit or harm. This visualization highlights the complex relationship between patient‐perceived outcomes in epilepsy treatment. Blue represents the patients that experienced an apparent worsening of seizures by at least 25%, but received a true ≥ 10% reduction from the seizure frequency they would have had without the ASM. Orange represents the patients that experienced an apparent ≥ 50% reduction in seizures, but the medication truly reduced their seizure by < 10% of what they would have been without the ASM. (The outliers with greater than 500% increase in seizure frequency are consistent with real world data from regulatory trials). Outliers with 100% apparent ASM reduction are shown in the upper right, consistent with the small fraction of seizure free patients reported in ASM trials.

Among the 100,000 seizure diaries extracted from CHOCOLATES, 29,962 synthetic patients met the trial inclusion criteria. The pattern of responses in our 10,000 simulated trials using these patients achieved a response distribution consistent with those reported in adjunctive ASM regulatory studies [3]. Among the 10,000 trials, the mean percentage of patients with an increase in SF of any degree (> 0%) was 21% on ASM and 36% on placebo. (Figure 2). Those on simulated ASM experienced a median reduction from baseline of 36% when taking an ASM compared to 17% on placebo. The 50% responder rate was 35% on ASM and 20% on placebo. When taking an ASM, 12% of the synthetic patients had apparent significant worsening (≥ 25% increase). Despite experiencing a significant increase, 76% of these patients were true responders. (Figure 3) What is more, only 4% of the patients that were apparent worseners were true worseners. Lastly, 12% of the apparent responder patients were true nonresponders.

## 4 Discussion

Our findings suggest that the overwhelming majority of patients with a concerning increase in SF actually received an ASM benefit. All these patients received greater benefit than a small minority of the apparent responders patients who actually did not have any beneficial ASM (Figure 3). This highlights the complexity of assessing ASM efficacy based solely on observed changes in SF from baseline. The natural fluctuations in SF in our model masked the true efficacy of the medication. Clinically, these patients are at an increased risk of being labeled as ASM nonresponders in an outpatient setting. This misclassification may result in restricted treatment options and unnecessary exposure to alternative treatments with less favorable side effect profiles.

Clinicians and patients cannot definitively know the counterfactual when trying a new ASM. This makes it challenging to justify ongoing treatment in the face of apparent worsening. Continuing an ASM under these circumstances may expose patients to unnecessary side effects without clear evidence of efficacy and could potentially strain the therapeutic relationship. There are psychological factors at play as well. For example, if a patient was willing to take on the risks of side effects in the hopes of going from 6 seizures per month to 3 seizures per month but ended up with 9 seizures per month, then the psychological let‐down could be far greater compared to the same increase from 6 to 9 in the absence of a new ASM.

Additionally, identifying if there are heterogeneous treatment effects and which individuals receive a true benefit within a trial setting is difficult [9] and requires a multiple crossover design [10]. This would increase the overall exposure time to placebo. The field is moving toward trial designs that reduce placebo exposure due to the concerns of higher risks of sudden unexpected death in epilepsy in the placebo group [11]. That said, in the clinical setting our results offer reassurance regarding the potential utility of retrying “failed efficacy” ASMs. This decision may spare the patient from ASMs with more challenging side effects. There is potential to retrospectively test the results of our paper by examining the percentage of patients that have found benefit after reexposure to a previously “failed” ASM. If the retrospective data are in accordance with our results, then our conclusions could be studied in a prospective manner.

It is important to recognize that as a simulation study, all the results presented here remain to be validated with clinical data. However, the key insights revealed here are derived from the extensive variability in seizure frequency that occurs in the CHOCOLATES simulator. The simulator was designed to reflect statistical features observed across multiple clinical datasets, including heterogeneity in SF, the L‐relationship of SF, multiple risk cycles, seizure clustering, and maximum seizure count. The “L‐relationship” reflects the observation seen across multiple datasets that there is a reliable connection between the log of the average seizure rate versus the log of the standard deviation of the same count, per patient [12]. This relationship appears to be true in self‐reported seizure diaries, physician‐curated seizure diaries, wearable device‐based seizure annotations [13], and intracranial EEG‐based seizure diaries. Therefore, even if some of the subtle modeling features of CHOCOLATES are imperfect, the variability seen in clinical datasets is already known to be approximated by this simulator.

Constraining our simulation to ASM trial parameters not only gives us confidence that our synthetic patients resemble real‐world ASM‐trial patients, but it also limits us to response rates seen during the 12‐week maintenance phase. In this context, our choice to define the minimally effective benefit as a 10% median reduction over placebo, while seemingly modest, is the observed benefit of the commonly prescribed 1000 mg/day of levetiracetam during the maintenance phase in the regulatory efficacy trial [8].

Even though our model produces many of the statistical features seen in seizure diaries, the use of synthetic data may not capture all the nuances of individual patient experiences [10]. Lastly, the assumption of a Gaussian‐distributed medication effect with a mean reduction of 20% and a standard deviation of 10% may not reflect the true unknown interindividual variability seen with ASMs. Alternative models exist, yet we either find the same basic conclusions or the extreme heterogeneous effects assumptions produce results that do not reflect real‐world data (see Appendix).

## 5 Conclusion

Our simulation study demonstrates that natural variability in SF can lead to misclassification of ASM effectiveness when starting a new ASM results in higher SF. These findings highlight the complexity of assessing ASM efficacy based solely on changes from baseline and underscore the potential for natural fluctuations to mask true treatment effects.

Our results suggest that clinically retrying previously failed ASMs could be beneficial for those with limited treatment options, especially when side effect profiles are favorable. Further research is needed to validate these findings in clinical settings and to develop strategies that account for natural variability in seizure frequency when assessing treatment responses.

## Author Contributions

C.N.H. and D.M.G. contributed to the conception and design of the paper, analysis and interpretation of the data. C.N.H. completed the necessary coding for the work.

## Conflicts of Interest

D.M.G. is an unpaid advisor for Epilepsy AI and Eysz. He has been a paid advisor for Magic Leap and has received speaker fees from AAN, AES, ACNS, and AI in Epilepsy and Neurology. He previously served as a paid consultant for Neuro Event Labs, IDR, LivaNova, and Health Advances and has received grants from NIH and BIDMC. None of these relationships are believed to represent a direct conflicts of interest for the present work, but they are disclosed in the spirit of transparency. C.N.H. declares no conflicts of interest.

## Supporting information


Appendix

## Data Availability

Our simulations were conducted using R (version 4.4.0) as well as Python (v 3.12.4). We utilized ChatGPT‐4.o (OpenAI, California, USA) to draft an initial coding framework and to debug portions of the R code. The tool provided assistance intermittently between July‐December 2024. C.N.H. verified that the code was accurate and appropriately handled the data to ensure the reliability of the results. Open source code is available at https://github.com/forkcandles/misalignment_project.

## References

[1] R. F. M. Chin , W. O. Pickrell , F. Guelfucci , M. Martin , and R. Holland , “Prevalence, Healthcare Resource Utilization and Mortality of Lennox‐Gastaut Syndrome: Retrospective Linkage Cohort Study,” Seizure 91 (2021): 159–166. 34161904 10.1016/j.seizure.2021.05.025

[2] D. M. Goldenholz , R. Moss , J. Scott , S. Auh , and W. H. Theodore , “Confusing Placebo Effect With Natural History in Epilepsy: A Big Data Approach,” Annals of Neurology 78 (2015): 329–336. 26150090 10.1002/ana.24470 PMC4546516

[3] C. Henry , M. Creasey , L. Cannon , S. W. Terman , and D. Goldenholz , “Increased Seizure Frequency Graphs in Medication Trials: FDA Labels vs. Peer‐Review,” Seizure 131 (2025): 29–34. 40480038 10.1016/j.seizure.2025.05.021 PMC12279039

[4] D. M. Goldenholz and M. B. Westover , “Flexible Realistic Simulation of Seizure Occurrence Recapitulating Statistical Properties of Seizure Diaries,” Epilepsia 64, no. 2 (2023): 396–405, 10.1111/epi.17471. 36401798 PMC9905290

[5] E. Ben‐Menachem and V. Biton , “Efficacy and Safety of Oral Lacosamide as Adjunctive Therapy in Adults With Partial‐Onset Seizures,” Epilepsia 48 (2007): 1308–1317. 17635557 10.1111/j.1528-1167.2007.01188.x

[6] J. A. French , M. Mosier , S. Walker , K. Sommerville , and N. Sussman , “A Double‐Blind, Placebo‐Controlled Study of Vigabatrin Three g/Day in Patients With Uncontrolled Complex Partial Seizures. Vigabatrin Protocol 024 Investigative Cohort,” Neurology 46 (1996): 54–61. 8559421 10.1212/wnl.46.1.54

[7] J. A. French and G. L. Krauss , “Evaluation of Adjunctive Perampanel in Patients With Refractory Partial‐Onset Seizures: Results of Randomized Global Phase III Study 305,” Epilepsia 54 (2013): 117–125. 22905857 10.1111/j.1528-1167.2012.03638.x

[8] S. D. Shorvon , A. Löwenthal , D. Janz , E. Bielen , and P. Loiseau , “Multicenter Double‐Blind, Randomized, Placebo‐Controlled Trial of Levetiracetam as Add‐On Therapy in Patients With Refractory Partial Seizures,” European Levetiracetam Study Group Epilepsia 41 (2000): 1179–1186. 10.1111/j.1528-1157.2000.tb00323.x 10999557

[9] A. Hopkins , P. Davies , and C. Dobson , “Mathematical Models of Patterns of Seizures. Their Use in the Evaluation of Drugs,” Archives of Neurology 42 (1985): 463–467. 3888154 10.1001/archneur.1985.04060050061009

[10] S. Senn , K. Rolfe , and S. A. Julious , “Investigating Variability in Patient Response to Treatment‐A Case Study From a Replicate Cross‐Over Study,” Statistical Methods in Medical Research 20 (2011): 657–666. 20739334 10.1177/0962280210379174

[11] W. T. Kerr and L. Y. Ngo , “Time to Prerandomization Seizure Count Design Sufficiently Assessed the Safety and Tolerability of Perampanel for the Treatment of Primary Generalized Tonic‐Clonic Seizures,” Epilepsia 65 (2024): 2412–2422, 10.1111/epi.18023. 38864472 PMC11325753

[12] D. M. Goldenholz , S. R. Goldenholz , R. Moss , et al., “Is Seizure Frequency Variance a Predictable Quantity?,” Annals of Clinical Translational Neurology 5 (2018): 201–207. 29468180 10.1002/acn3.519 PMC5817844

[13] B. Zhang , W. V. Chen , G. Regalia , D. M. Goldenholz , and R. W. Picard , “Statistical Characteristics of Large‐Scale Objective Tonic‐Clonic Seizure Records From Medical Smartwatches Used in Daily Life,” Epilepsia 65 (2024): 3255–3264. 39287615 10.1111/epi.18109 PMC11573641

