# Supplementary material for: The Road Not Taken: Misclassifying an Anti‐Seizure Medication as a Failure
Source: Ann Clin Transl Neurol. 2025 Jul 21;12(10):2130–5. doi: 10.1002/acn3.70139 (PMC12516234; doi:10.1002/acn3.70139)
Supplement: Supplementary file 1 — Appendix [file ACN3-12-2130-s001.docx]

**Appendix**

**Medication Effect**

We reviewed the median reduction difference between placebo and anti-seizure medication (ASM) in adjunctive ASM regulatory trials. We elected a 20% reduction effect. It is known that some individuals benefit significantly greater than others and some epilepsies, such as sodium channelopathies, can be worsened by certain ASM. Therefore, we elected to apply a heterogeneous effect rather than a homogeneous effect across patients. Appendix Figure 1 shows the various standard deviations we considered. We chose a standard deviation of 10% to allow some individuals to be truly worsened by the ASM while recognizing that is likely a rare occurrence. A standard deviation of 10% leads to the ASM causing the SF to increase in 2% of patients. Observational data estimates that ASMs cause exacerbations in 1.3%.[^1^](https://paperpile.com/c/fcKpyq/iqQ3) Therefore we rejected standard deviations above 10% that assume the ASM is the direct cause of seizure frequency increases in a sizable percentage of the population. Additionally, there are strong arguments to model homogeneous effects due the assumptions required to discover meaningful heterogeneous effects.[^2,3^](https://paperpile.com/c/fcKpyq/EizwK+zrJP5) Therefore we elected for a modest standard deviation. We explored the heterogeneous assumptions analytically (Appendix Table 1) and through simulation (Appendix Figure 1 and 2 and Appendix Table 2).

(Appendix Table 1)

| Percentage of patients where the ASM directly causes increase of seizures  $P(X>1) = 1-\phi\left( \frac{1-\mu}{\sigma} \right)$  $\phi$ is the CDF of a normal random variable | |
| --- | --- |
| Standard Deviation | Percent harmed by ASM |
| 0.05 | 0 |
| 0.10 | 2 |
| 0.15 | 9 |
| 0.20 | 16 |
| 0.25 | 21 |
| 0.30 | 25 |
| 0.35 | 28 |


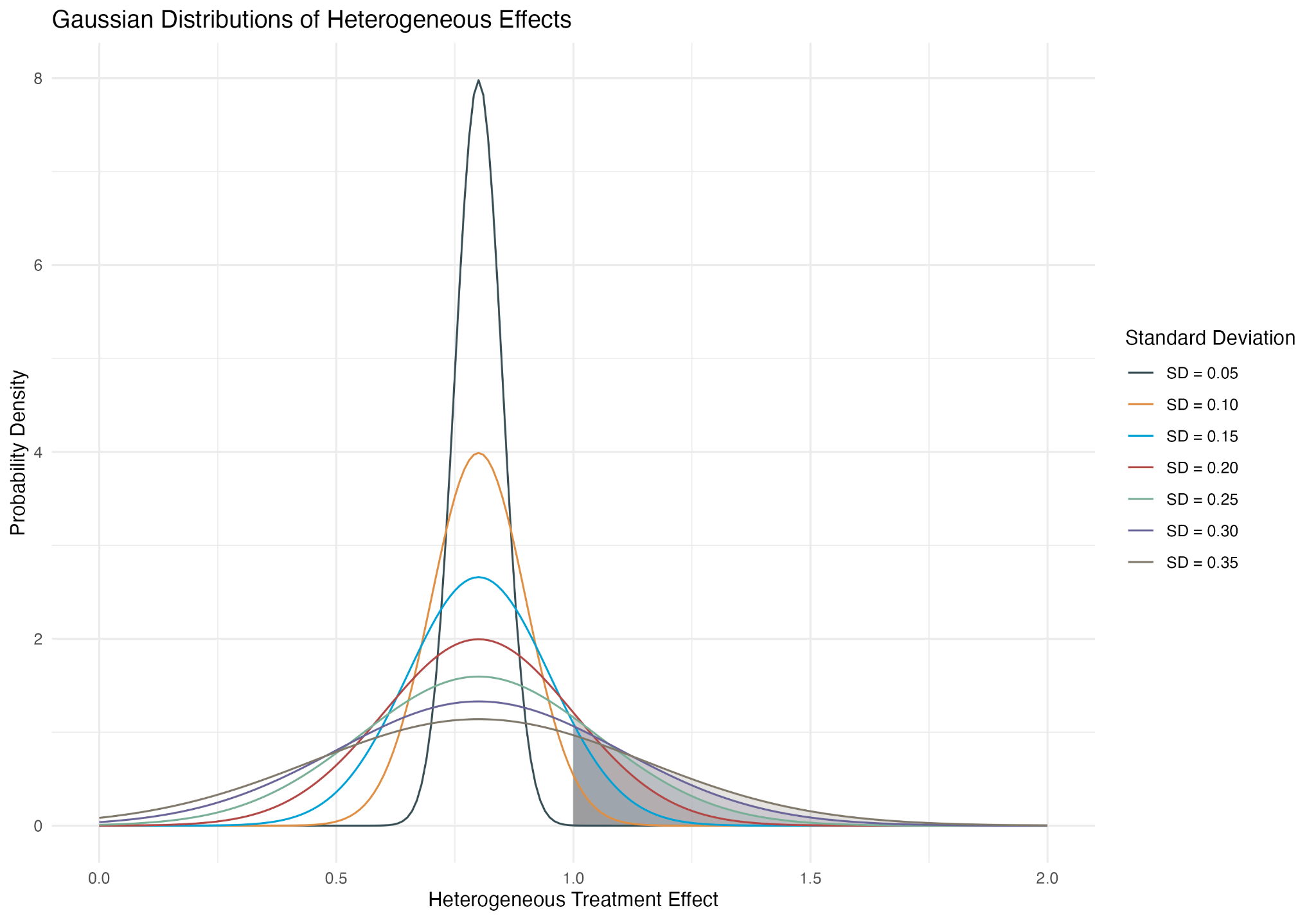


Appendix Figure 1: Heterogeneous mean effect of 20% reduction with various standard deviations. The shaded region signifies the amount of harm by the ASM based on assumptions of the standard deviation of the heterogeneous effect.


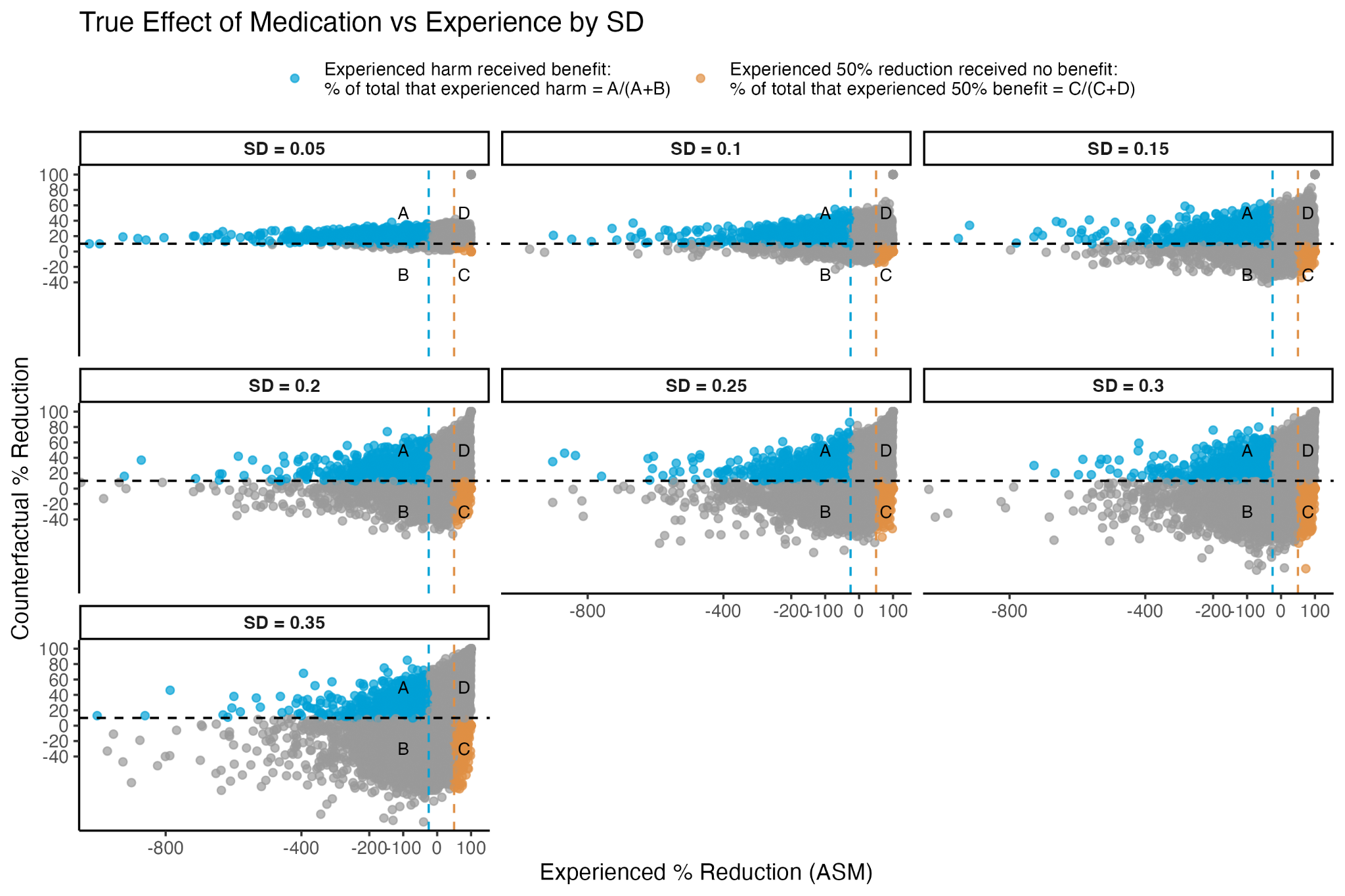


Appendix Figure 2: The distribution of benefit and harm among the 29,962 synthetic patients when applying the treatment effect seen in Appendix Figure 1 by various standard deviations in treatment heterogeneity. The blue dashed line is the 25% increase in seizure frequency. The orange line is the 50% reduction in seizure frequency.

| Appendix Table 2: Implications of the degree of treatment heterogeneity from simulated data | | | | | |
| --- | --- | --- | --- | --- | --- |
| SD | % ASM Harm | % with >25% increase in seizures and ASM directly causes increase | % with > 25% increase in seizures | % with > 25% increase in seizures that received benefit | % with > 50% reduction that did not receive benefit |
| 0.05 | 0 | 0 | 12 | 97 | 4 |
| 0.10 | 2 | 4 | 12 | 76 | 12 |
| 0.15 | 9 | 18 | 13 | 59 | 16 |
| 0.20 | 15 | 32 | 14 | 47 | 17 |
| 0.25 | 21 | 46 | 14 | 38 | 17 |
| 0.30 | 24 | 53 | 15 | 32 | 16 |
| 0.35 | 28 | 62 | 16 | 26 | 16 |

CHOCOLATES does not accurately capture seizure freedom. Therefore, seizure freedom was modeled based on probabilities assigned to each patient, with probabilities varying according to treatment conditions and seizure frequency. When the patient was not taking an ASM their baseline seizure freedom probability was uniformly randomized between 0-2%. When they were taking an ASM their seizure freedom was based on their seizure frequency. For patients with a lower seizure frequency (fewer than 20 seizures), this probability was randomized between 2% and 8%. For patients with higher seizure frequencies, the probability of seizure freedom with ASM remained at the baseline level. Studies support the idea that those with lower seizure frequency have a higher probability of achieving seizure freedom.[^4^](https://paperpile.com/c/fcKpyq/Z98Jh) Once each patient had a baseline probability for seizure freedom then another a random probability was generated for each patient, and if it was less than the assigned chance of seizure freedom, the patient was classified as seizure-free; otherwise, they were classified as not seizure-free.

Below are the trials we cross-referenced for seizure freedom rates and then checked to see if our simulated trials generated the same seizure freedom rates.

Perampanel 2.3% (8 mg), 5.0% (12 mg) versus placebo 1.5% [^5^](https://paperpile.com/c/fcKpyq/aOPkh)

Perampanel 2.2% (8 mg), 1.5% (12 mg) versus placebo 0.0% [^5^](https://paperpile.com/c/fcKpyq/aOPkh)

Levetiracetam 5.0% (1000 mg), 2.0% (2000 mg) versus placebo 0.9% [^6^](https://paperpile.com/c/fcKpyq/nGOkr)

Levetiracetam 3.0% (1000 mg), 8.0% (3000 mg) versus placebo 0.0% [^7^](https://paperpile.com/c/fcKpyq/l4yOf)

Lacosamide 0.9% (200 mg), 4.6% (400 mg), 1% (600 mg) versus 0.0% placebo [^8^](https://paperpile.com/c/fcKpyq/NCfYs)

Lacosamide 2.0% (400 mg), 5.2% (600 mg) versus 0% placebo [^9^](https://paperpile.com/c/fcKpyq/0Jwf7)

**Model Checking**

If simulated trials from the synthetic patients with our simulated heterogeneous medication effect closely replicate results seen in adjunctive ASM regulatory trials, then we can be more confident that the synthetic patients provide insights into how SF variability can lead to misclassification of treatment failure of real world patients. As seen in Appendix Figure 2 the median reduction differences between ASM and placebo, when the standard deviation is 0.1, are similar to regulatory trials. Appendix Figure 3 shows the distribution of percent change from baseline of 4 randomly selected trials of the 10,000 trials with 200 patients in each group. Both the median reduction distributions and percent change distributions were consistent with results seen in adjunctive ASM regulatory trials.

*Extreme values and seizure freedom*

Figure 3 in the main manuscript shows outliers with SF increases greater than 500%. There is minimal data on these outliers as most publications lump individuals into broad categories of increases greater than 0, 25, 50 or 100%. However, where the data is available these types of outliers are present and do not appear to be an error with the model. The zonisamide prescribing label shows individuals with increases in excess of 500%, the regulatory trial for cannabidiol showed individuals with 337% increases from baseline, and the FDA clinical review document for rufinamide showed that the range of increased SF went up to a 1,190% increase for tonic-atonic seizures for individuals taking rufinamide.[^10–12^](https://paperpile.com/c/fcKpyq/86qV0+aNnrW+VK8U5)

In Figure 3 in the main manuscript, a distinct cluster of individuals achieving seizure freedom appears in the top right corner, clearly separated from the main distribution. This departure likely reflects limitations in using percent change as an outcome measure, especially when considering seizure freedom from a counterfactual perspective. Percent change alone does not fully encapsulate the concept of seizure freedom. The true medication effect will always be 100% even if an individual’s baseline was 4 seizures/month and was naturally going to decrease to 1 seizure/month without an ASM. By taking the ASM the individual achieved seizure freedom. The natural fluctuation decreased the SF by 75%, but the ASM was 100% effective against that last seizure. Some might argue that the medication effectiveness is a 25% reduction from baseline. However, the medication does not act on the baseline, it only acts on the potential seizures (ie the counterfactual scenario) once the individual is taking the medication.

This becomes clearer if we consider a scenario where the individual's baseline was 4 seizures per month and would have naturally increased to 6 without ASM but instead reached seizure freedom with the ASM. Here, the ASM provides a 100% reduction relative to both the baseline and the counterfactual. Using the baseline as the benchmark might suggest a nonsensical 150% reduction in seizure frequency, as it implies a negative seizure count—an impossible outcome. These complexities result in a simplified cluster that visually stands apart from the rest of the graph, underscoring how counterfactual considerations and natural variability impact not only our interpretation of medication effects but also the limitations of our chosen outcome measures to capture what is truly going on.


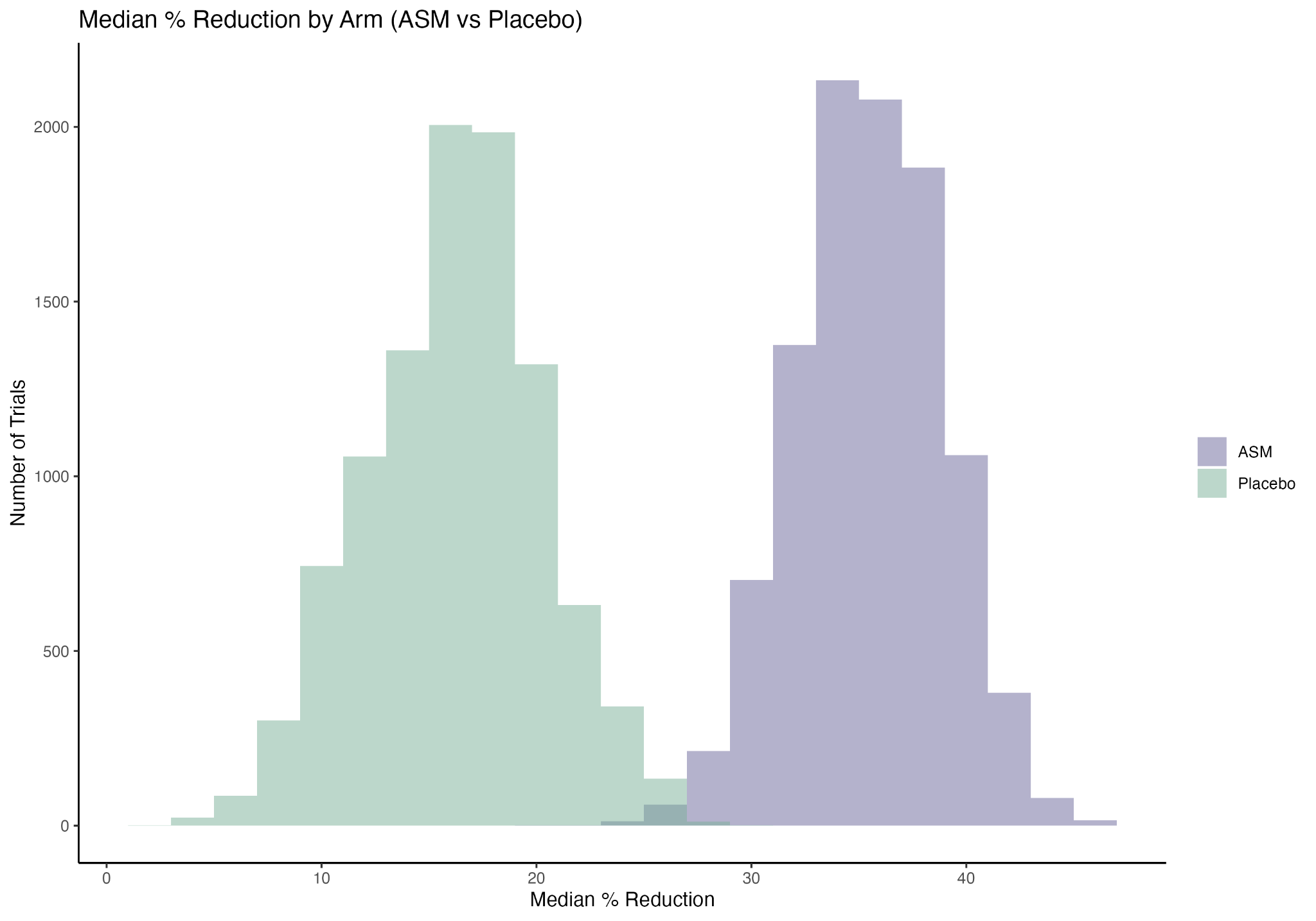


Appendix Figure 3: Histograms of 10,000 trials of size N = 400 showing the median percent reduction between ASM and placebo


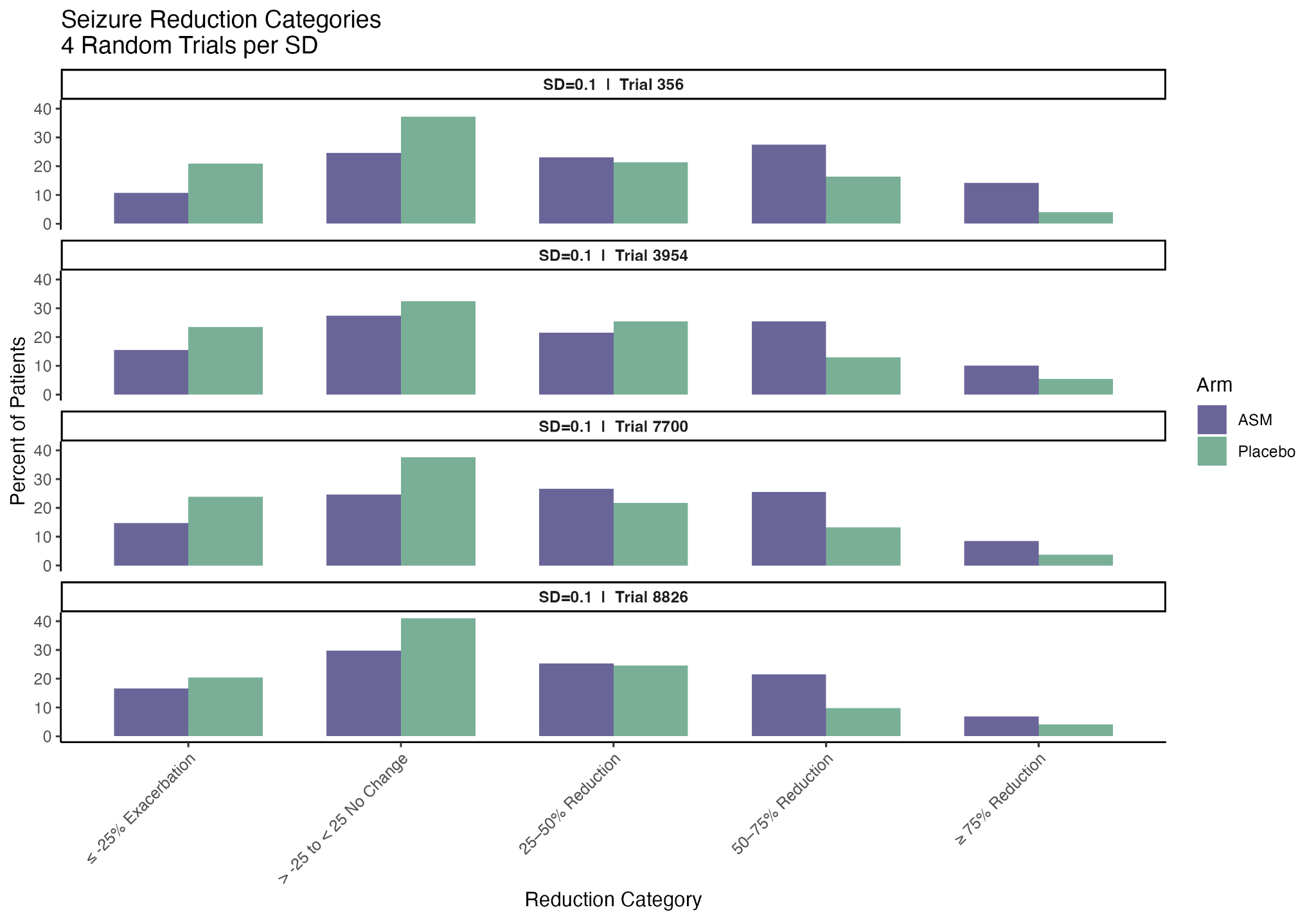


Appendix Figure 4: Histograms percent change distributions from baseline of 4 randomly selected from the 10,000 simulated trials.

1. [Jaramillo, M. A., Pham, T., Kamrudin, S., Khanna, R. & Maheshwari, A. Seizure exacerbation with anti-seizure medications in adult patients with epilepsy. *Epilepsy Res.* **181**, 106885 (2022).](http://paperpile.com/b/fcKpyq/iqQ3)

2. [Senn, S., Rolfe, K. & Julious, S. A. Investigating variability in patient response to treatment--a case study from a replicate cross-over study. *Stat. Methods Med. Res.* **20**, 657–666 (2011).](http://paperpile.com/b/fcKpyq/EizwK)

3. [Senn, S. Individual response to treatment: is it a valid assumption? *BMJ* **329**, 966–968 (2004).](http://paperpile.com/b/fcKpyq/zrJP5)

4. [Lattanzi, S. *et al.* Adjunctive brivaracetam and sustained seizure frequency reduction in very active focal epilepsy. *Epilepsia* **64**, 2922–2933 (2023).](http://paperpile.com/b/fcKpyq/Z98Jh)

5. [French, J. A. *et al.* Evaluation of adjunctive perampanel in patients with refractory partial-onset seizures: results of randomized global phase III study 305. *Epilepsia* **54**, 117–125 (2013).](http://paperpile.com/b/fcKpyq/aOPkh)

6. [Shorvon, S. D., Löwenthal, A., Janz, D., Bielen, E. & Loiseau, P. Multicenter double-blind, randomized, placebo-controlled trial of levetiracetam as add-on therapy in patients with refractory partial seizures. European Levetiracetam Study Group. *Epilepsia* **41**, 1179–1186 (2000).](http://paperpile.com/b/fcKpyq/nGOkr)

7. [Cereghino, J. J. *et al.* Levetiracetam for partial seizures: results of a double-blind, randomized clinical trial: Results of a double-blind, randomized clinical trial. *Neurology* **55**, 236–242 (2000).](http://paperpile.com/b/fcKpyq/l4yOf)

8. [Ben-Menachem, E. *et al.* Efficacy and safety of oral lacosamide as adjunctive therapy in adults with partial-onset seizures. *Epilepsia* **48**, 1308–1317 (2007).](http://paperpile.com/b/fcKpyq/NCfYs)

9. [Chung, S. *et al.* Lacosamide as adjunctive therapy for partial-onset seizures: a randomized controlled trial. *Epilepsia* **51**, 958–967 (2010).](http://paperpile.com/b/fcKpyq/0Jwf7)

10. [Amdipharm Limited. Dublin 9, Ireland. *Zonegran® (zonisamide)*.](http://paperpile.com/b/fcKpyq/86qV0) <https://www.accessdata.fda.gov/drugsatfda_docs/label/2020/020789s036lbl.pdf> [(Revised: 04/2020).](http://paperpile.com/b/fcKpyq/86qV0)

11. [Devinsky, O. *et al.* Trial of Cannabidiol for Drug-Resistant Seizures in the Dravet Syndrome. *N. Engl. J. Med.* **376**, 2011–2020 (2017).](http://paperpile.com/b/fcKpyq/aNnrW)

12. [Ohidul Siddiqui, Kun Jin, James Hung, Norman Hershkowitz, Courtney Calder. *Statistical Review and Evaluation*.](http://paperpile.com/b/fcKpyq/VK8U5) <https://www.accessdata.fda.gov/drugsatfda_docs/nda/2008/021911s000_StatR.pdf> [(2005).](http://paperpile.com/b/fcKpyq/VK8U5)
